# Psycho-social correlates of leisure-time physical activity (LTPA) among older adults: a multivariate analysis

**DOI:** 10.1186/s11556-020-00238-6

**Published:** 2020-03-04

**Authors:** Sunwoo Lee, Chungsup Lee, Jaesung An

**Affiliations:** 1 Faculty of Physical Culture, Palacký University Olomouc, Třída Míru 117, 771 11 Olomouc, Czech Republic; 2 Department of Recreation and Leisure Studies, California State University, Long Beach, CA 90840 USA; 3 Department of Recreation, Sport and Tourism, University of Illinois at Urbana-Champaign, Champaign, IL 61820 USA

**Keywords:** Leisure-time physical activity (LTPA), Psycho-social correlates, Positive mindset, Physical activity promotion

## Abstract

**Background:**

Still, a considerable number of older adults hardly meet the daily physical activity recommendation. The current study examined how the elderly’s attitudinal and perceptional aspects were associated with their Leisure-Time Physical Activity (LTPA) participation in order to provide insight into the physical activity promotion.

**Methods:**

Study used a total sample of 10,700 older adults aged 65+ drawn from the Health and Retirement Study (HRS) 2014–2015 in USA. Multiple questionnaire items were employed to assess older adults’ attitudes and outlook across different life domains. LTPA participation was measured using two indicators—walking and sports/exercise. A technique of adjusted multivariate analysis was employed to examine the relationships between predictors and outcome variables.

**Results:**

Results indicated that psychosocial indicators were significantly associated with older adults’ LTPA: self-perception of ageing, self-efficacy, intrinsic motivation, spiritual engagement, perceived social tie and neighbourhood safety; while measures of perceived social relations and neighbourhood safety demonstrated different associations with walking and sports/exercise on its intensity.

**Conclusions:**

Health professionals should be well-informed about psychosocial roles, either as facilitators or constraints, in older adults’ physical activity participation. Incorporation of psychosocial intervention into physical activity promotion can help older adults develop positive attitudes and inner strength linked to their health behaviour.

## Introduction

Benefits of Leisure-Time Physical Activity (LTPA) in older adulthood are well-documented [1–3]. However, older adults are the least physically active of all age groups, and only small portion of older adults meet the physical activity recommendation [4, 5]. For example, at best 30% of American older adults, aged between 60 and 69, were satisfied with recommended physical activity guideline and less than 10% of the older adults, aged 70 and over, barely met the physical activity guideline [6]. Older adults rather desist from their favourite pastime physical activities due to declined physicality and psychological instability [7–9]. Psychological constraints such as a loss of control, fear of falling or injury, and lack of companionship often lead to low morale, less competence and interest in physical activities in older adults [10, 11]. That is, public health professionals in physical activity interventions ought to better help older adults strengthen inner capability to be engaged in physical activity.

Existent literature shows that optimistic mindset and intrinsic motivation positively contribute to older adults’ daily coping strategies, health improvement, and psychological well-being [12–14]. Self-efficacy, positive perception of ageing, and positive social interactions play an important role in helping older adults more engage in health behaviours such as healthy eating and physical activity participation [11, 15–17]. Older adults’ involvement and perceived benefits of physical activities also depend on the favourable perception of the neighbourhood environment [18–20]. All of this suggests that the relations between psycho-social and physical activity need to be closely considered to motivate older adults to more participate in LTPA. However, only a handful studies examined different psychosocial aspects en bloc and its association with LTPA among older adults.

Building on this, the current study examined psychosocial correlates of older adults’ LTPA employing seven psychosocial factors that represent an individual’s cognitive and affective states across different life domains: self-perception of ageing, self-efficacy, intrinsic motivation, perceived social relations, spiritual engagement, optimism, and perceived neighbourhood safety. We hypothesized that psychosocial variables would significantly influence different level of physical activity participation among older adults. Findings of our study will serve as a basis for a long-term and sustainable physical activity intervention strategies by manipulating more effectively different psychosocial influences in the elderly’s physical activity participation.

## Methods

### Study design and sample

This study employed a secondary data analysis using data released from the Health and Retirement Study (HRS) 2014–2015 in USA. Initial raw dataset involved a total of 18,714 individuals aged between 18 and 105 years old, and comprised information about the respondents’ physical and mental health (e.g., cognitive function, physical measures and biomarkers), life trajectories (e.g., family structure, job history, retirement, divorce, widowhood), social capital (e.g., pension/insurance, housing), and survey data collected using self-administered leave-behind questionnaire that assessed respondents’ psychosocial aspects of everyday life and well-being. Of respondents, we retained those aged 65 years or older for the current study. This left a final sample of 10,700 participants ranging in age from 65 to 105 years old at the point of data collection (Mean = 76.32, *SD* = 7.74). The sample comprised 41.0% males and 59.0% females. Caucasians accounted for 80.0, 15.0% were Black or African Americans, and 5.0% were others. Among respondents, 9.9% considered themselves Hispanics. Among the respondents, 53.6% were married, 12.6% were separated or divorced, 30.1% were widowed, and 3.4% reported they were never married. Nearly half of the sample (49.1%) maintained a high school diploma, 16.3% had two or four-year college degree, and 9.5% had master or professional degree. It is important to note that Research Ethics Board of the authors’ institutions waived the requirement for ethics review because the current study employed publicly available secondary data.

### Measurement

#### Predictor variables

Both single and multiple Likert-scale items were used to examine different psychosocial domains: ageing perception, self-control, motivational force, perceived social relations, spiritual/religious engagement, optimism, and perceived neighbourhood safety.

A total number of three questionnaire items were used to measure self-perception of ageing (e.g., “As I get older, things are better than I thought they would be”). Respondents were asked how they feel about their age and about the things that happened, as they get older. Using a 7-point Likert scale, they were to select an answer ranging from 1 “strongly disagree” to 7 “strongly agree.”

Three questionnaire items were used to measure self-control over different life domains (e.g., health, social life, and financial issues). The respondents were asked to rate an amount of control, using a 0 to 10 scale where 0 means “no control at all” and 10 means “very much control.”

Motivational force was assessed using a single questionnaire item (e.g., “I am willing to take risks during leisure and sport”). The respondents were asked to indicate how willing they were to take risks during leisure and sports, using a 0 to 10 scale that 0 means “unwilling to take any risks” and 10 means “fully prepared to take risks.”

Perceived social tie was measured using a total of 5 questionnaire items (e.g., “There are people I feel close to”). The respondents were asked to indicate how much of the time they feel about their relationship with people around, using a 3-point Likert scale, from 1 “hardly ever or never” to 3 “often.”

Spiritual/religious engagement was measured using four different questionnaire items (e.g., “I try hard to carry my religious beliefs over into all my other dealings in life”). Using a 6-point Likert scale, respondents were to select an answer ranging from 1 “strongly disagree” to 6 “strongly agree.”

Optimism was measured using a total of three indicators (e.g., “I expect more good things to happen”). Using a 6-point Likert scale, they were to select an answer ranging from 1 “strongly disagree” to 6 “strongly agree.”

Perceived neighbourhood safety was measured using a single questionnaire item that asked respondents how they felt about their local area that is everywhere within a 20-min walk or about a mile of their home. The respondents were asked to indicate the level of agreement with the statement, “feeling safe walking alone in this area after dark,” ranged from 1 “least satisfied” to 10 “most satisfied.”

#### Outcome variables

From the HRS survey questionnaire, two questionnaire items were employed in order to measure Leisure-time Physical Activities (LTPA). Study respondents were asked to indicate how often they played sports or exercise and how often they walked for 20 min or more. Using a 7-point Likert scale, respondents selected an answer, ranging from 1 “never” to 7 “once a week.” We considered playing sports/exercising and walking as two different dependent variables.

### Data analysis

A final sample data was prepared based on series of data scanning including univariate outliers examination and normality tests. Univariate outliers were detected by computing z-scores in the distribution. Because we used large sample sizes (> 100), acceptable cut-off range of 4.0 to - 4.0 was used. Scatter plots were examined in order to confirm the linearity of the sample data [21, 22]. Kolmogorov-Smirnov (K-S) normality test was used to assess the distribution of the sample data. According to the results, no normality issues were identified with the study variables.

Descriptive analysis and correlational test were performed. To ensure scale reliability for the multiple-item scales (i.e., ageing perception, self-control, perception of social relationship, spiritual/religious engagement, and optimism), a test of composite reliability was performed. We created a composite index of the multiple-item measure by summing scores across different measured variables. Based on weighted sample, we employed multivariate analysis using General Linear Modeling technique in order to examine study hypotheses [22]. Covariates in the analyses included major predictor variables and demographic variables including gender, age, race/ethnicity, marital status, and education level. A list-wise deletion of cases was used to handle missing data while performing multivariate analysis. IBM Statistic software (SPSS 20.0) was used throughout the procedures of the data preparation and analysis.

## Results

### Descriptive and correlational analyses

Table 1 provides descriptive analysis of the measured variables (mean and standardized deviations) and internal consistency of the study constructs. The measures which used multiple questionnaire items had adequate to excellent internal reliability; the Cronbach’s alpha scores ranged from .771 (self-control) to .937 (spiritual/religious engagement).

**Table 1:** Descriptive statistics and internal consistency of the measured variables

Variable(s)	Items	α	Mean	SD
Predictor variables^a^				
Ageing perception	happy as when younger	.803	3.76	1.69
	better than thought		3.99	1.57
	satisfied with ageing		4.41	1.44
Self-control	amount of control over health	.771	7.04	2.45
	control over social life		7.78	2.37
	control over financial situation		7.54	2.53
Motivational force^c^	prepared to take risks during leisure and sport	–	3.39	2.76
Perceived social relationship	people I can talk to	.861	2.55	.61
	people I can turn to		2.56	.60
	people understand me		2.41	.62
	people I feel close to		2.61	.57
	feel part of group of friends		2.30	.72
Spiritual/religious engagement	believe in god	.937	5.28	1.50
	events unfold by a divine plan		4.80	1.62
	try hard to carry out beliefs		4.87	1.60
	find strength in religion		5.04	1.57
Optimism	optimistic about own future	.812	4.44	1.37
	expect the best in uncertain times		4.33	1.29
	expect more good things to happen		4.78	1.28
Perceived neighbourhood safety^c^	feel safe to walk alone after dark	–	5.28	1.78
Dependent variables^b,c^				
Often play sport/exercise		–	3.57	2.27
Often walk for 20 mins		–	4.18	2.19

^a^ Ageing perception measure used a 7-point Likert-type scale, 1 (strongly disagree) to 7 (strongly agree); Self-control was rated on a 10-point Likert-type scale, 0 (no control at all) to 10 (very much control); Motivational force for leisure was rated on a 10-point Likert-type scale, 0 (unwilling to take any risks) to 10 (fully prepared to take risks); Measure of social tie used a 3-point Likert-type scale, 1 (hardly ever or never) to 3 (often); Spiritual/religious engagement and optimism used a 6-point Likert-type scale,1 (strongly disagree) to 6 (strongly agree); Perceived neighbourhood safety was rated on a 10-point Likert-type scale, 1 (least satisfied) to 10 (most satisfied)

^b^ Dependent variables used a 7-point Likert-type scale, 1 (never) to 7 (once a week)

^c^ Due to a single questionnaire item used, no internal consistency was examined

We also found the nine study factors were weakly to moderately correlated with each other (−.033 to .497). The correlation coefficients are well below the .60 maximum between any two variables, suggested as a cut-off for the multivariate analysis to avoid multicollinearity problem [21]. In addition, the measures of two dependent variables were not largely correlated (*r* = .497) to proceed with multivariate test. Table 2 summarizes the correlations among study variables.

**Table 2:** Correlations between study variables

	Ageing perception	Self-control	Motivational force-leisure	Perceived social tie	Spiritual engagement	Optimism	Neighbourhood safety	Sport/exercise	Walking
	(AP)	(SC)	(MF)	(ST)	(SPE)	(OP)	(NS)	(S-E)	(WK)
AP	–								
SC	.409^**^	–							
MF	.118^**^	.124^**^	–						
ST	.322^**^	.306^**^	.032^*^	–					
SPE	.086^**^	.057^**^	−.083^**^	.109^**^	–				
OP	.366^**^	.279^**^	.075^**^	.305^**^	.140^**^	–			
NS	.115^**^	.127^**^	.043^**^	.148^**^	−.061^**^	.089^**^	–		
S-E	.185^**^	.195^**^	.213^**^	.151^**^	−.057^**^	.121^**^	.108^**^	–	
WK	.212^**^	.216^**^	.111^**^	.122^**^	−.033^*^	.102^**^	.061^**^	.497^**^	–

** p < .01, * p < .05

### Multivariate effects of psychosocial variables

Multivariate tests (here Pillai’s Trace) were significant except one predictor, optimism (see Table 3). There was a statistically significant difference in LTPA among the older adults based on ageing perception, *F* (2, 3622) = 36.174, *p* < .001; self-control, *F* (2, 3622) = 41.686, *p* < .001; motivational force, *F* (2, 3622) = 56.820, *p* < .001; perceived social tie, *F* (2, 3622) = 6.908, *p* < .01; spiritual/religious engagement, *F* (2, 3622) = 8.338, *p* < .001; and neighbourhood safety, *F* (2, 3622) = 4.770, *p* < .01. These findings supported existent literature that psychosocial domains are significantly associated with LTPA among older adults. However, we found no statistically significant difference in LTPA based on optimism.

**Table 3:** Significant multivariate effects^a^

Predictor variable(s)	Pillai’s trace	*F*	*df*	Error *df*	Sig.	Hypothesis test
Ageing perception	.020	36.174	2.00	3622.00	.000	supported
Self-control	.023	41.686	2.00	3622.00	.000	supported
Motivational force	.030	56.820	2.00	3622.00	.000	supported
Perceived social relationship	.004	6.908	2.00	3622.00	.001	supported
Spiritual/religious engagement	.005	8.338	2.00	3622.00	.000	supported
Optimism	.001	1.607	2.00	3622.00	.201	*not supported*
Neighbourhood safety	.003	4.770	2.00	3622.00	.009	supported

^a^ Dependent variables: Sports/exercise and walking 20 min

Based on the significant multivariate effects on dependent variables, we further provide information about how the psychosocial variables are associated with each dependent variable, walking 20 min and playing sports/exercise (see Table 4). It is important to note that we removed the measure of optimism from the analysis due to the non-significant multivariate effect on dependent variables. We found the measure of sports/exercise was significantly influenced by six predicting variables: ageing perception, *F* (1, 3623) = 31.268, *p* < .001; self-control, *F* (1, 3623) = 46.692, *p* < .001; motivational force, *F* (1, 3623) = 112.944, *p* < .001; perceived social tie, *F* (1, 3623) = 13.183, *p* < .001; spiritual/religious engagement, *F* (1, 3623) = 14.750, *p* < .001; and neighbourhood safety, *F* (21, 3623) = 9.541, *p* < .01.

**Table 4:** Significant multivariate effects on dependent variable(s)

Variable(s)		*df*	Error *df*	*F*	Sig.
Ageing perception	Sports/exercise	1	3623	31.268	.000
	Walking 20 min	1	3623	68.248	.000
Self-control	Sports/exercise	1	3623	46.692	.000
	Walking 20 min	1	3623	72.542	.000
Motivational force	Sports/exercise	1	3623	112.944	.000
	Walking 20 min	1	3623	16.932	.000
Social tie	Sports/exercise	1	3623	13.183	.000
	Walking 20 min	1	3623	.914	.339
Spiritual/religious engagement	Sports/exercise	1	3623	14.750	.000
	Walking 20 min	1	3623	8.976	.003
Neighbourhood safety	Sports/exercise	1	3623	9.541	.002
	Walking 20 min	1	3623	1.896	.169

Measure of walking 20 min was also significantly influenced by ageing perception, *F* (1, 3623) = 68.248, *p* < .001; self-control, *F* (1, 3623) = 72.542, *p* < .001; motivational force, *F* (1, 3623) = 16.932, *p* < .001; and spiritual/religious engagement, *F* (1, 3623) = 8.976, *p* < .01. However, those two measures related to social-environmental factors, positive social tie and neighbourhood safety, had no statistically significant effects on walking 20 min. This is discussed further in the section that follows.

According to the significant multivariate test, our study hypotheses were supported to a great extent. The majority of independent variables had a statistically significant effect on both sports/exercise and walking 20 min: ageing perception, self-control, motivational force for leisure activity, and spiritual/religious engagement. Significant multivariate effects were also found for the measures of perceived social relations and neighbourhood safety on sports/exercise participation.

## Discussion

The current study aimed at better understanding of psychosocial correlates of LTPA among older adults. Study findings are consistent with the existent literature that older adults’ physical activity participation is significantly associated with their psychosocial manifestation [11, 15]. Results indicated that those older adults who demonstrated positive ageing perception, self-control, and interest in leisure and sport, were more likely to engage in LTPA. Crombie et al. [8] show that a lack of motivation and interest is the most influential factor that impedes older adults’ LTPA over the lacking fitness or physical infrastructure. A study by Orsega-Smith et al. [17] also provided evidence that self-efficacy of perceived physical capability was significantly related to LTPA among older adults aged over 60 years. This led us to argue that we ought to focus more on older adults’ inner struggles and potential to facilitate their motivation in sport and physical activity.

Our finding is in line with previous studies that perceived social relationships and neighbourhood safety significantly related to sport/exercise participation [19, 23]. However, multivariate analysis indicated that older adults’ walking activity was not significantly associated with better social relationship and neighbourhood safety. Walking, as one of the most favourable moderate physical activities in older adults, might be less affected by social and environmental factors. For those older adults, a deeper understanding of ageing, a sense of independence, and positive bonds to their social and physical environments might be an intangible resource that helps them to maintain healthy behaviours and lifestyle. We ought to provide suitable care and support for those older adults who feel vulnerable and hesitant to participate in sport/exercise on a regular basis or other recommended moderate physical activity.

It was an interesting finding that there was no significant multivariate effect of optimism, while the other positive attitudinal variables were found to have significant associations with LTPA. Literature provides few possible explanations. First, it might be because older adults’ optimistic view does not necessary lead to actual behavioural decision. We note that optimism tends to regard as a predisposed personality trait in literature [24]. Therefore, optimism might play as a facilitator of better ageing perception, self-efficacy, and positive social relationships rather than as a direct link to physical activity participation. Second, those older adults with a higher sense of optimism might be easily content or better adapt to life change with age. They would unconsciously prefer declining such activities that cause physical challenge and difficulties. Although our study data did not provide empirical evidence of the relationship between optimism and LTPA, we still concede that there might be positive association between optimism and health-related behaviours. Rasmussen et al. [13] examined 83 empirical studies and reported there was a significantly positive relationship between optimism and physical health outcomes. We suggest that a longitudinal study design and meditating model test will help determine if those elderly with optimistic view would better maintain positive psychological domains and how it affects health and well-being over time.

Health education and practice should be better informed about psychosocial contribution to older adults’ LTPA as a means to alter and increase their health beliefs and behaviours. For example, psychotherapeutic intervention (e.g., meditation practice) and easily accessible counselling programs (e.g., leisure counselling and retirement planning programs) both individual and group-level can help the elderly persons to maintain independency, positive and proactive mindset, and spiritual actualization. Indeed, studies examining the effectiveness of mindfulness-based intervention programs show that older adults who practice mindfulness meditation significantly reduce loneliness and chronic pains [25]. Also, recreation and physical activity interventions aimed at seniors should develop and coordinate re-adjusted sports and exercise (i.e., less intense forms and practice) so older adults can continue participating in their favoured physical activity. More structured community-based program, including opportunity for social events (e.g., a group Nordic walking) and neighbourhood maintenance for safety can compensate for a lack of motivation and interest in physical activity among the elderly.

Lastly but also importantly, some limitations should be addressed. The current cross-sectional study was developed as a preliminary investigation of psychosocial influence on older adults’ LTPA using HRS sample data. A series of companion studies will be further developed along with our study limitations. First, we acknowledge that cross-sectional nature of sample data is insufficient to explain direct cause and effect between study variables. There might be a variability of the relationships between psychosocial factors and older adults’ physical activity. For example, one of the predicting variables in the current study, perceived social relationships, can be considered as outcome of LTPA. Employing path model test will help to better clarify the relationships between predictor and outcome variables.

Second, future studies should consider test for model specification to examine if psychosocial correlates of LTPA among older adults would be changed by any intervening variables. Several background variables, such as gender, age group (young, old, and oldest), and ethnicity should be taken into account in the model. Also, literature shows that older adults’ physical activity is closely related to their physical capability and environment [26]. Therefore, if the study would involve expanded psychosocial domains in analysis such as physical activity-related beliefs and self-rated health, it would help more precisely predict the psychosocial antecedent of older adults’ LTPA. Future studies also develop a longitudinal model to determine if psychosocial correlates of older adults’ LTPA are consistent over time in a same group of study respondents.

## Conclusion

Our findings highlight the important role of positive psychological capital in older adults’ healthy lifestyle and behaviour; the intra-personal psychological variables (i.e., motivational force, self-efficacy, and positive ageing perception) were the critical contributors to the LTPA engagement among older adults. This led us to argue that we ought to develop and provide enduring psychological resources of self-reliance, hope, and resilience to better promote physical activity intervention for ageing population. Our study also reveals that psychosocial influence might vary according to the different type of LTPA. Results indicated that sport/exercise engagement was related to most of the psychosocial variables, while recreational walking was significantly associated with inner-oriented values rather than perceived social and environmental factors. This suggests that we ought to consider complexity of psychosocial influence on older adults’ LTPA engagement.

## Data Availability

The datasets used and/or analyzed during the current study are available from the corresponding author on reasonable request.
